# [Retracted] Atorvastatin increases lipopolysaccharide-induced expression of tumour necrosis factor-α-induced protein 8-like 2 in RAW264.7 cells

**DOI:** 10.3892/etm.2026.13197

**Published:** 2026-06-02

**Authors:** Ming-Wei Liu, Mei-Xian Su, Wei Zhang, Li Wang, Chuan-Yun Qian

Exp Ther Med 8:219–228, 2014; DOI: 10.3892/etm.2014.1722

Following the publication of the above paper, it was drawn to the Editor's attention by a concerned reader that certain of the western blot data featured in Figs. 1B, 6 and 9A, and mRNA expression data included in Figs. 3 and 4 were strikingly similar to data that had already been published in other articles in different journals that were written by different authors at different research institutes.

After independently analyzing the data in this paper, the Editorial Office were able to verify the reader’s concerns. Owing to the fact that the contentious data in the above article were found to be strikingly similar to data that had already been published elsewhere in other papers in different journals, the Editor of *Experimental and Therapeutic Medicine* has decided that this paper should be retracted from the Journal. The authors were asked for an explanation to account for these concerns, but the Editorial Office did not receive a reply. The Editor apologizes to the readership for any inconvenience caused.

